# Optical and nuclear imaging of glioblastoma with phosphatidylserine-targeted nanovesicles

**DOI:** 10.18632/oncotarget.8763

**Published:** 2016-04-16

**Authors:** Víctor M. Blanco, Zhengtao Chu, Kathleen LaSance, Brian D. Gray, Koon Yan Pak, Therese Rider, Kenneth D. Greis, Xiaoyang Qi

**Affiliations:** ^1^ Division of Hematology-Oncology, Department of Internal Medicine, University of Cincinnati College of Medicine, Cincinnati, Ohio 45267, USA; ^2^ Division of Human Genetics, Department of Pediatrics, Cincinnati Children's Hospital Medical Center, Cincinnati, Ohio 45229, USA; ^3^ Department of Radiology, University of Cincinnati College of Medicine, Cincinnati, Ohio 45267, USA; ^4^ Molecular Targeting Technologies, Inc., West Chester, Pennsylvania 19380, USA; ^5^ Department of Cancer Biology, University of Cincinnati College of Medicine, Cincinnati, Ohio 45267, USA

**Keywords:** glioblastoma, liposome, PET, optical imaging, SapC-DOPS

## Abstract

Multimodal tumor imaging with targeted nanoparticles potentially offers both enhanced specificity and sensitivity, leading to more precise cancer diagnosis and monitoring. We describe the synthesis and characterization of phenol-substituted, lipophilic orange and far-red fluorescent dyes and a simple radioiodination procedure to generate a dual (optical and nuclear) imaging probe. MALDI-ToF analyses revealed high iodination efficiency of the lipophilic reporters, achieved by electrophilic aromatic substitution using the chloramide 1,3,4,6-tetrachloro-3α,6α-diphenyl glycoluril (Iodogen) as the oxidizing agent in an organic/aqueous co-solvent mixture. Upon conjugation of iodine-127 or iodine-124-labeled reporters to tumor-targeting SapC-DOPS nanovesicles, optical (fluorescent) and PET imaging was performed in mice bearing intracranial glioblastomas. In addition, tumor vs non-tumor (normal brain) uptake was compared using iodine-125. These data provide proof-of-principle for the potential value of SapC-DOPS for multimodal imaging of glioblastoma, the most aggressive primary brain tumor.

## INTRODUCTION

**Figure 1:**
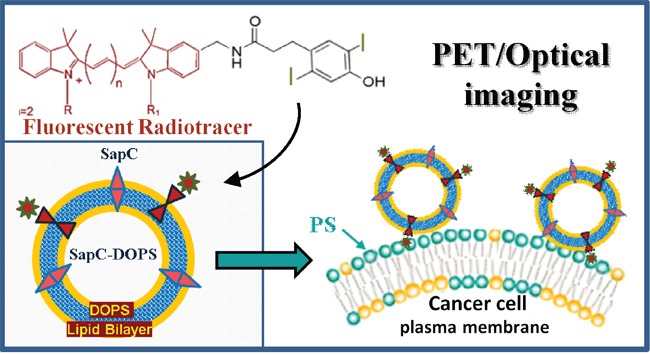
Schematic representation of phenol-substituted, iodinated lipophilic dyes conjugated to tumor-targeted SapC-DOPS nanovesicles for dual imaging of glioblastoma Tumor selectivity is determined by the affinity of SapC towards PS, a phospholipid sequestered in the inner leaflet of the plasma membrane in non-tumor cells, but markedly externalized upon neoplastic transformation.

Non-invasive imaging techniques such as radiographs and computed tomography (CT), Ultrasonography, Magnetic Resonance Imaging (MRI), diffuse optical tomography (DOT), and nuclear imaging modalities such as Positron Emission Tomography (PET) and Single-photon Emission Computed Tomography (SPECT) are invaluable diagnostic and monitoring tools for pathological processes like cancer. Frequently aided by contrast imaging agents, these structural and functional techniques rely on intrinsic physicochemical differences between tumors and the surrounding tissues, such as water density and the interstitial diffusion of fluids and macromolecules. As these properties are not exclusive of tumors, a key obstacle still hampering the usefulness of such modalities is the limited specificity (and to a lesser extent, sensitivity) of those approaches to unambiguously detect and identify tumor processes. These caveats can potentially be overcome by integrating tumor-targeted molecular markers and dual (optical/nuclear, nuclear/MRI, etc.) imaging probes that combine the strengths of individual imaging modalities [1–3]. One such tumor marker is phosphatidylserine (PS), a membrane phospholipid that resides in the inner (cytosolic) leaflet of the plasma membrane of normal, healthy cells, but becomes exposed in the outer surface in many viable tumor and tumor-associated vascular (endothelial) cells [4–10]. Exploiting this property, work in our laboratory led to development of SapC-DOPS, a biologic anticancer agent consisting of a human protein, saposin C (SapC), embedded into the lipid bilayer of unilamellar nanovesicles composed of dioleoylphosphatidylserine (DOPS). SapC is a naturally occurring membrane protein that binds PS with high affinity and activates lysosomal enzymes [11, 12]. By targeting PS-rich domains on neoplastic cell membranes, SapC-DOPS has been shown to selectively initiate lipid-mediated pathways that lead to lysosomal destabilization and necrosis on glioblastoma cells [13–15] or to apoptosis, secondary to ceramide accumulation, as in neuroblastoma, pancreatic, skin and lung cancer cells [16–20]. The potential of SapC-DOPS for cancer imaging has been reported using optical and MRI techniques in several solid tumor models, including glioblastoma [21–23]. In this study, we describe the synthesis of monomolecular, phenol-substituted membrane intercalating lipophilic dyes, and a simple iodination procedure that allows the generation of dual (optical/nuclear) imaging probes (Figure 1). By conjugating these probes to tumor-targeted SapC-DOPS nanovesicles, we provide proof-of-principle for the use of this nanoparticle-based system for dual imaging of glioblastoma, the most aggressive and prevalent brain tumor.

## RESULTS

### Synthesis of phenol substituted lipophilic fluorochromes

**Figure 2:**
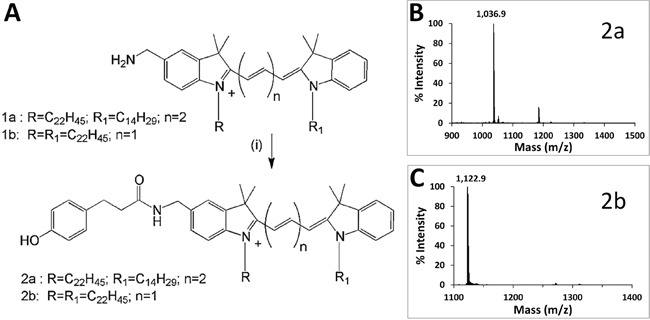
Synthesis and characterization of phenol-substituted dye analogs **A.** Synthetic scheme to prepare phenol substituted fluorescent analogs. **B.** MALDI-ToF spectrum of compound 2a. **C.** MALDI-ToF spectrum of compound 2b.

Phenol-substituted analogs of DiD (compound 2a, Figure 2) and DiI (compound 2b, Figure 2) amenable to direct electrophilic aromatic iodination were synthesized by Molecular Targeting Technologies, Inc. (MTTI; West Chester, PA) in one step from previously prepared compounds 1a and 1b [24], as described in Figure 2A. Compounds 2a and 2b were analyzed by mass spectrometry (Figures 2B and 2C) and NMR (Supplementary Figure 1) to confirm their structures. The far-red fluorochrome present in DiD (compound 1a) (ε = 225,000 M^−1^cm^−1^at 650 nm) is an indodicarbocyanine suitable for optical imaging with 633 nm HeNe or 647 nm Kr-Ar lasers and standard Cy5 filters (Ex ~ 650 nm; Em ~ 670 nm) [25]. Reduced autofluorescence in the far red provides improved sensitivity and allows optical imaging at greater depths within tissue [26] than is possible in the visible range. The lipophilic cationic dialkylcarbocyanine fluorophore DiI (compound 1b) (ε = 148,000 M^−1^cm^−1^ at 550 nm) exhibits orange fluorescence and is suitable for optical imaging with 543 HeNe or 561 diode lasers and standard Rhodamine/Cy3 filters (Em_545_/Ex_610_). DiI is highly fluorescent, well retained after incorporation into membranes and metabolically stable *in vivo*. The hydrocarbon tail configurations present in 2a and 2b have been shown to provide good incorporation efficiency into SapC-DOPS nanovesicles and allowed optical imaging of glioblastoma and metastatic brain tumors [13, 14].

### Iodination procedures

**Figure 3:**
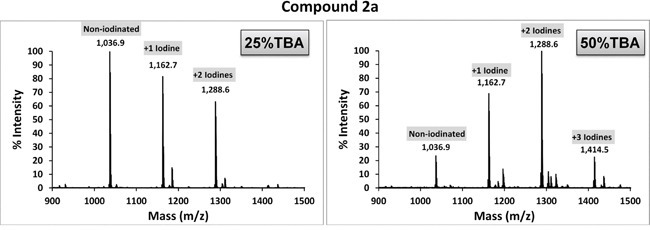
MALDI-ToF spectrum of compound 2a reacted with ^127^I Cold iodination with NaI (^127^I) was carried out by dissolving compound 2a in TBA/PBS and reacting the mixture with NaI in Iodogen pre-coated tubes. Higher labeling efficiency was achieved using a 1:1 co-solvent ratio (50% TBA).

**Figure 4:**
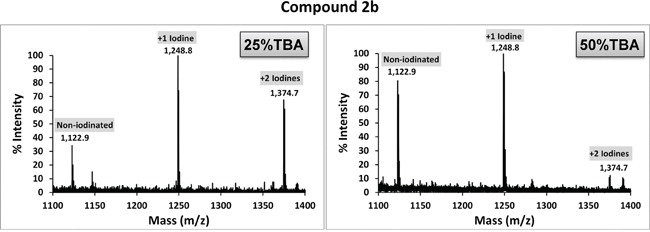
MALDI-ToF spectra of compound 2b reacted with ^127^I Iodination of compound 2b with NaI (^127^I) was carried out by dissolving compound 2b in TBA/PBS and reacting the mixture with NaI in Iodogen pre-coated tubes. Higher labeling efficiency was achieved using a 1:3 co-solvent ratio (25% TBA).

Compounds 2a and 2b were subjected to direct iodination with Iodogen (1,3,4,6-tetrachloro-3α,6α-diphenylglucoluril) [27], a mild oxidizing agent commonly used in the radioiodination of proteins [28, 29]. Since these dyes are insoluble in water, a number of solvents (chloroform, ethanol and DMSO) were initially evaluated, with unsatisfactory results. Successful incorporation of non-radioactive iodine (^127^I) was achieved by using tert-butyl alcohol (TBA; 2-methyl-2-propanol), a highly water-soluble tertiary alcohol. MALDI-ToF spectra for compounds 2a and 2b reacted with ^127^I (NaI) are shown in Figures 3 and 4, respectively. The ratio of TBA to PBS affected iodination efficiency: for 2a, the highest efficiency was attained with 50% TBA, while for 2b the best results were achieved with 25% TBA. Since a fraction of each dye remained unreacted (non-iodinated) under the conditions tested, employing different TBA:PBS ratios and/or allowing lengthier reaction times will likely improve the method's efficiency. Radioiodination of compound 2a was performed in a similar way, using ^125^I (Perkin Elmer, Boston, MA; 105 mCi/ml, carrier-free) or ^124^I (IBA Molecular, Dulles, VA; 100 mCi/ml, no-carrier-added) instead. In brief, 100 μg of compound 2a in TBA were reacted with 4.7 μl (500 μCi) ^125^INa plus 1 mM NaI, or with 25 μl (1.45 mCi) ^124^I in a final volume of 100 μl (TBA:PBS = 1:1). After 15-20 min incubation in Iodogen tubes, radiolabeled 2a was separated from free radioisotope through reverse phase filtration as described above. The radioiodine yield (percent of original activity present in eluted compound 2a) was ~ 37% for ^125^I and ~ 50% for ^124^I, as measured with a dose calibrator.

### Formulation of SapC-DOPS nanovesicles with iodinated fluorochromes

**Figure 5:**
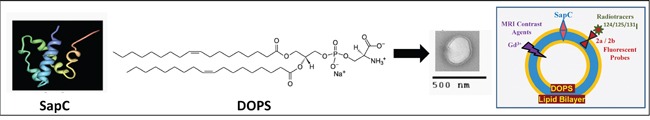
Structure of SapC-DOPS nanovesicles Under slightly acidic conditions, the hydrophobic protein SapC and the phospholipid dioleoyl-phosphatidylserine (DOPS) assemble into stable ~200 nm proteoliposomes, as seen by freeze-fracture electron microscopy. As depicted in the schematic illustration on the right, multimodal cancer imaging and therapy are possible by functionalization with contrast agents and radioligands.

The assembly of SapC-DOPS-(2a/2b) nanovesicles was carried out as previously described [19], by first combining DOPS and compound 2a, drying the organic solvents and adding SapC in the presence of a small volume of acidic (pH 5.0) citrate buffer. After addition of PBS, the mix is sonicated and finally passed through a gel filtration column to eliminate unconjugated fluorochrome. Assembled nanovesicles are unilamellar and have a mean diameter of 200 nm [22] (Figure 5).

### *In vivo* targeting and imaging of glioblastoma with SapC-DOPS coupled to radioiodinated fluorescent reporters

**Figure 6:**
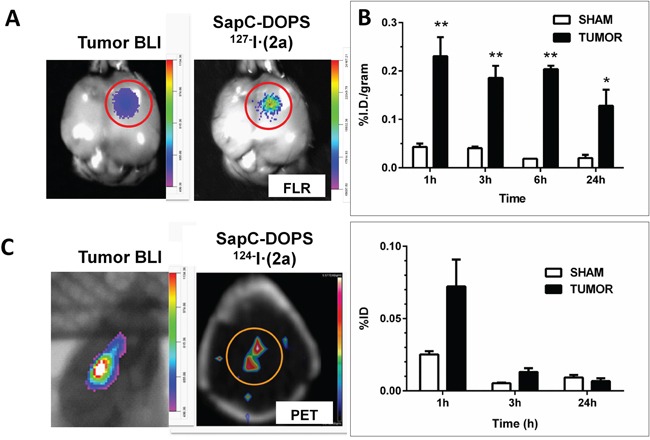
Selective targeting of intracranial glioblastoma by SapC-DOPS conjugated with an iodinated fluorochrome **A.** A mouse bearing a human glioblastoma xenograft (U87ΔEGFR-Luc cells) was injected (tail vein) with SapC-DOPS conjugated with cold-labeled, (^127^I) phenolic 2a. 24 h later tumor bioluminescence (BLI) and compound 2a's fluorescence (right) were assessed in the excised brain, confirming colocalization. **B.** Mice bearing intracranial glioblastoma (TUMOR) or saline (SHAM) were injected (tail vein) with SapC-DOPS conjugated with ^125^I-labeled phenolic 2a (5 ± 0.2 μCi). At different time points, tissues and organs were dissected and the incorporated radioactivity was measured and expressed as % injected dose (ID)/gram. Top graph shows brain activity; bottom graph shows thyroid organ activity. Tumor bearing mice: n = 7 (1 h); n = 6 (3 h); n = 2 (6 h); n = 6 (24 h). Sham: n = 4 (1, 3, 24 h); n = 2 (6 h). *, p < 0.05; **, p < 0.01 (t-test). **C.** microPET imaging of a glioblastoma in a mouse brain 24 h after administration of two i.v. injections (spaced 2 h apart) of 300 μl (50 μCi) SapC-DOPS-^124^I· (2a) nanovesicles. A CT scan was acquired for anatomical co-registration and attenuation correction of the PET data. Imaging data was processed using Siemens IRW software (v4.1). Concurrent bioluminescence imaging (BLI) confirmed the presence of glioblastoma.

Using both optical imaging [13, 14, 16, 19]] as well as MRI [22, 23], we have shown that SapC-DOPS nanovesicles efficiently and selectively target solid tumors, including glioblastoma, in living mice [13–15, 30]. Negligible tumor targeting was observed with non-targeted DOPS nanovesicles [13]. As proof of principle for the use of SapC-DOPS coupled to phenol substituted, radiolabeled fluorochromes for imaging of glioblastoma, we first tested whether the tumor-targeting ability of SapC-DOPS-(2a) was preserved upon iodination of the phenol group in the 2a reporter. We used an orthotopic glioblastoma mouse model to inject via tail vein SapC-DOPS-^127^I· (2a) and monitored its accumulation in the brain 24 h later. Figure 6A shows an example of these results, which suggest that the tumor-selective capacity of radioiodinated SapC-DOPS nanovesicles is similar to that of nanovesicles labeled with the non-radioactive, parent compound [13, 14].

Next, we assessed the tumor targeting properties and biodistribution profile of SapC-DOPS-^125^I· (2a). Iodine-125 has a long half-life (60 days) and low γ emission (35.5 keV), which is readily detected and yet prevents excessive radiation exposure, and has been applied with success in the treatment of gliomas [31, 32]. Nude mice with orthotopic human glioblastoma or without tumor (sham; intracranial saline injection) were injected i.v. with SapC-DOPS-^125^I· (2a) and sacrificed at 1, 3, 6 or 24 h. Brains were dissected, weighed, and tumor and brain radioactivity was measured with a gamma counter. ^125^I activity in tumors was low (< 1% ID/g) but significantly higher (> 4 fold) than in sham brains at all time points (Figure 6B). High activity was detected in the liver and spleen while moderate activity was present in the lungs (Supplemental Figure 2), reflecting extraction by the reticuloendothelial system. These data are in line with our previous studies showing transient (~ 48 h) accumulation of fluorescently labeled SapC-DOPS in these organs [19]. Low uptake (≤ 1%) was also detected in the heart, stomach, pancreas and kidneys at 24 h. Blood clearance was fast, as < 1% ID/ml was detected after 1 h. Thyroid activity was low (< 0.1% of injected dose) at all time points, indicating minimal release of free iodine (Figure 6B, bottom panel). More detailed studies need to be carried out to assess whether liver excretion of unbound radioiodinated fluorochrome contributes to the relatively large volume of distribution observed, which may reflect impaired stability of the nanovesicles upon conjugation with the reporter [33].

### PET imaging of glioblastoma using SapC-DOPS-^124^I· (2a)

Finally, we labeled compound 2a with ^124^I and assessed the potential of SapC-DOPS-^124^I· (2a) conjugates for microPET imaging of intracranial tumors. ^124^I is a relatively ‘long-lived’ (half-life of 4.2 days) positron emitter, which unlike commonly used, short-lived radio nuclides (^18^F,^11^C), allows for prolonged evaluation of residence time of the radionuclide within the tumor [34, 35] and extended assessment of the pharmacokinetics of SapC-DOPS. In addition, PET/CT with ^124^I can be used clinically to estimate tumor-absorbed dose for pre-therapy dosimetry prior to implementation of (^125^I)/(^131^I)-radiochemical therapies [36]. Figure 6C provides an example of a successful attempt to visualize an intracranial glioblastoma in a mouse *in vivo*.

## DISCUSSION

As early detection greatly improves the outcome in many types of cancer, the development of dual- or multimodal-imaging agents is highly desirable inasmuch as they combine the advantages of individual approaches, increasing efficacy and reducing imaging times [2, 37, 38]. For both bioimaging and therapy, nanoparticle-based compounds are very attractive as they offer a large surface area and high cargo capacity, are biologically inert or biocompatible, are fairly stable and possess well defined physicochemical properties (optical, magnetic, thermal, pH reactivity, etc.) that can be exploited by means of intrinsic (e.g. tumor pH) or extrinsic (e.g. light, radiation, magnetic force) stimuli [39–41]. While most nanoparticle systems will accumulate in solid tumors through a passive mechanism reliant on the ‘enhanced permeability and retention (EPR) effect’ [42, 43], the ability to functionalize these structures with a variety of ligands allows endowing them with tumor selectivity or specificity. As therapeutic agents, compared with non-encapsulated drugs, nanoparticles may offer improved pharmacokinetics, longer circulation times, and a better toxicological profile. Due to this versatility, which makes it possible to combine imaging and therapeutic capabilities, nanoparticle-based systems are ideally suited as tools for diagnostic therapy, or ‘theranostics’ [44, 45].

Early studies using either entrapped or lipid-bound radioisotopes sought to characterize the stability and biodistribution of different liposomal formulations [46–51], and to assess their tumor-targeting capabilities [52–56]. Because the efficiency of radionuclide encapsulation is generally low, subsequent approaches have sought to improve conjugation methods and to expand the number of radioligands to advance liposome-based bioimaging and therapy [33, 57–59]. On the other hand, numerous dual-imaging (optical/MRI [60, 61], optical/nuclear [1, 62–65], MRI/nuclear [66, 67]) or tri-modal imaging probes that exhibit passive or active tumor targeting have been synthesized [3, 37, 41]. Among these, studies in both solid tumor models and patients evaluating a series of tumor-selective, radioiodinated (or fluorescent dye-coupled) phospholipid ether (alkylphosphocholine) analogs (e.g. CLR1404) revealed significant potential for these compounds for tumor imaging and therapy [68].

In this report we describe the synthesis of phenol-substituted, membrane-intercalating fluorescent reporters (compounds 2a and 2b) and a method by which they can be easily, rapidly, efficiently and inexpensively radioiodinated to be assembled into liposomes for combined fluorescence (optical) and nuclear (planar scintigraphy, PET, SPECT) tumor imaging. The use of commercially available Iodogen pre-coated tubes provides a simpler and faster method that compares favorably to other radioiodination methods such as lactoperoxidase and chloramine T [29]. Selectivity towards glioblastoma, the most prevalent and aggressive brain tumor in adults, is achieved by using SapC-DOPS nanovesicles, a theranostic agent with affinity towards PS, a phospholipid abundantly exposed on the surface of many cancer cells [16, 30, 69]. Studies showing that SapC-DOPS selectively targets both spontaneous and xenografted glioblastomas, as well as breast and human lung cancer-derived brain metastases in mice, have been published recently [13, 14, 23]. In addition to being amenable to fast, facile and economic iodination, membrane-intercalating dyes such as compound 2a and 2b might provide prolonged tumor retention when compared with some lipid-soluble chelators tested as liposome radiolabels [70, 71] [e.g. hexamethy-lpropylene-amine-oxime (HMPAO); N, N-bis(2-mercaptoethyl)-N', N'-diethylethylenediamine (BMEDA); 8-hydroxyquinoline (oxine); etc.] [72, 73]. Biodistribution of ^125^I-labeled nanovesicles showed a 4-8 fold higher uptake in glioblastoma as compared with sham brains, and very low thyroid uptake. This suggests selective tumor targeting and minimal reporter degradation in blood.

In this proof of concept study we chose to test ^124^I (half-life = 4.2 days) as it allowed more experimental flexibility and is suitable for microPET imaging in our facility. Nanovesicle conjugation with ^123^I (half-life = 13 h) for SPECT can be further tested, which may be preferable for differentiating tumor recurrence from radiation necrosis [74]. Optimization studies will seek to improve radiochemical yield and nanovesicle retention of the radio-iodinated compounds, a common concern in liposomal systems functionalized with high-energy radioisotopes [75]. In summary, the nanoparticle-based system presented in this report represents a potentially useful tool for both improved PET/SPECT imaging, and fluorescence-guided surgical resection of brain tumors. Future studies will also test the hypothesis that SapC-DOPS elicits enhanced antitumor effects upon conjugation with phenol-substituted reporters labeled with iodine-131 (^131^I), a low-cost, readily available and widely used therapeutic radionuclide, for bioradiotherapy of glioblastoma and other solid tumors.

## MATERIALS AND METHODS

### Synthesis of mono-phenol substituted fluorochrome analogs

Compound 1a was treated [Figure 2A (i)] with 3-(4-hydroxyphenyl) propionic acid (Sigma, St Louis) in the presence of N, N, N′, N′-Tetramethyl-O-(1H-benzotriazol-1-yl) uronium hexafluorophosphate (HBTU) in DMF containing triethylamine. After stirring at room temperature for 24 h, the mixture was concentrated and purified by silica gel chromatography eluting with increasing amounts of methanol (1% to 5%) in dichloromethane to furnish 2a (32% yield). Compound 2b was prepared from 1b in a similar fashion in 59% yield.

### Iodination procedures

Iodination with NaI was carried out using Iodogen pre-coated iodination tubes (Pierce, Rockford, IL) according to the manufacturer's suggested (direct) procedure with some modifications. For cold labeling procedures, 120 μg of compound 2a (or 100 μg of compound 2b) in tertiary butyl alcohol (TBA; 10 mg/ml stock solutions) was added to TBA in a glass test tube, and NaI (^127^I) in PBS was then added in a total reaction volume of 100 μl. TBA:PBS co-solvent ratios of 1:1 (TBA 50%) or 1:3 (TBA 25%) were tested. Final [NaI] was 1 mM. The mixture was then briefly vortexed, added to the iodination tube (Iodogen) -pre-rinsed with PBS- and incubated for 15 min at RT with periodic shaking. Labeled compounds were recovered by elution through Sep-Pak (C4 reverse phase) columns by sequential addition of 1 ml methanol, 1 ml PBS, iodinated sample, 1 ml PBS, and 0.3 ml 2:1 chloroform:methanol. The eluate was vacuum-dried and resuspended in 100 μl ethanol. To verify the degree of mass shift consistent with iodination status, samples were diluted1:5, 1:10, and 1:20 in MALDI matrix (5 mg/mL α-cyano- hydroxy cinnamic acid in 10 mM ammonium phosphate, 60% ACN/0.1% formic acid) and spotted for MALDI-ToF analysis. All spectra were acquired in positive ion Reflector Mode on a Sciex 4800 MALDI-TOF/TOF Instrument.

Radioiodination of phenolic 2a was performed as described above, replacing NaI with ^125^I (Perkin Elmer, Boston, MA; 105 mCi/ml, carrier-free) or ^124^I (IBA Molecular, Dulles, VA; 100 mCi/ml, no-carrier-added). To this end, 100 μg of compound 2a in TBA were reacted with 4.7 μl (500 μCi) ^125^INa, or with 25μl (1.45 mCi) ^124^I, in a final volume of 100 μl (TBA:PBS = 1:1). After 15-20 min incubation in Iodogen tubes, radiolabeled 2a was separated from free radioisotope through reverse phase filtration as described above. The radioiodine yield (percent of original activity present in eluted 2a) was ~ 37% for ^125^I and 50% for ^124^I, as measured with a dose calibrator.

### SapC-DOPS nanovesicle preparation

25 to 40 μl of compound 2a/2b, iodinated and purified as described above, were mixed with 82 μg of DOPS (Avanti Polar Lipids, Alabaster, AL) in a glass tube and the mix solvent evaporated under nitrogen gas. Human recombinant SapC protein [11] (0.4 mg) was added to the DOPS-iodinated 2a film along with 20 μl citrate/phosphate buffer (pH 5.0). The addition of acidic buffer allows incorporation of SapC into the lipid phase. 1 ml PBS was then added and the mix was sonicated for 30 min at 4°C. SapC-DOPS-2a/2b nanovesicles were separated from non-conjugated fluorochrome using Sephadex G25 columns (PD-10; Amersham Pharmacia Biotech, Piscataway, NJ). Radioactivity of conjugated nanovesicles was measured using a Captus 3000 (Capintec) well counter. The stability of SapC-DOPS nanovesicles has been assessed in previous publications [16, 23, 76].

### Biodistribution studies

Orthotopic glioblastoma xenografts were produced by stereotactic injection of 1 × 10^5^ U87ΔEGFR-Luc cells into anesthetized female athymic nude mice, 2 mm lateral to bregma, at a depth of 3 mm [13]. Sham-operated animals received a saline injection. Once *in vivo* bioluminescence indicated tumor growth (~11 days post implantation) mice were injected via tail vein with SapC-DOPS-^125^I· (2a) (150 μl; 6.03 ± 0.02 μCi) and sacrificed at 1, 3, 6 or 24 h afterwards by cervical dislocation. Tissues (brain, lung, heart, thyroid, spleen, liver, stomach, pancreas, kidneys, muscle and fat) were removed, weighed, and placed in vials for radioactivity measurements in a gamma counter (2480 WIZARD^2^, Perkin Elmer). Blood activity was also measured.

### microPET imaging

After tumor growth was verified per *in vivo* bioluminescence imaging, two i.v. injections (300 μl; 2 h apart) with radioiodinated SapC-DOPS (50-100 μCi, 1.8-3.7 MBq, ^124^I) were administered. *In vivo* PET/CT imaging was performed 24 h later in a mouse anesthetized with 1-2% isoflurane, with body temperature being maintained for the duration of the imaging procedure with a warming pad. Low magnification, low resolution computed tomography (CT) images, calibrated in Hounsfield units, were first acquired for anatomical co-registration and PET data attenuation correction. PET images were acquired for 45 min using a Siemens Inveon MM (Knoxville, TN) tri-modal scanner. PET data was processed with an OSEM2D reconstruction algorithm using Siemens IRW 4.1 software.

## SUPPLEMENTARY FIGURES



## Abbreviations

SapC: Saposin C
DOPS: dioleylphosphatidylserine
PS: phosphatidylserine
