# Supplementary material for: Optical and nuclear imaging of glioblastoma with phosphatidylserine-targeted nanovesicles
Source: Oncotarget. 2016 Apr 16;7(22):32866–75. doi: 10.18632/oncotarget.8763 (PMC5078058; doi:10.18632/oncotarget.8763)
Supplement: Supplementary file 1 [file oncotarget-07-32866-s001.pdf]

Optical and nuclear imaging of glioblastoma with phosphatidylserine-targeted nanovesicles

SUPPLEMENTARY FIGURES

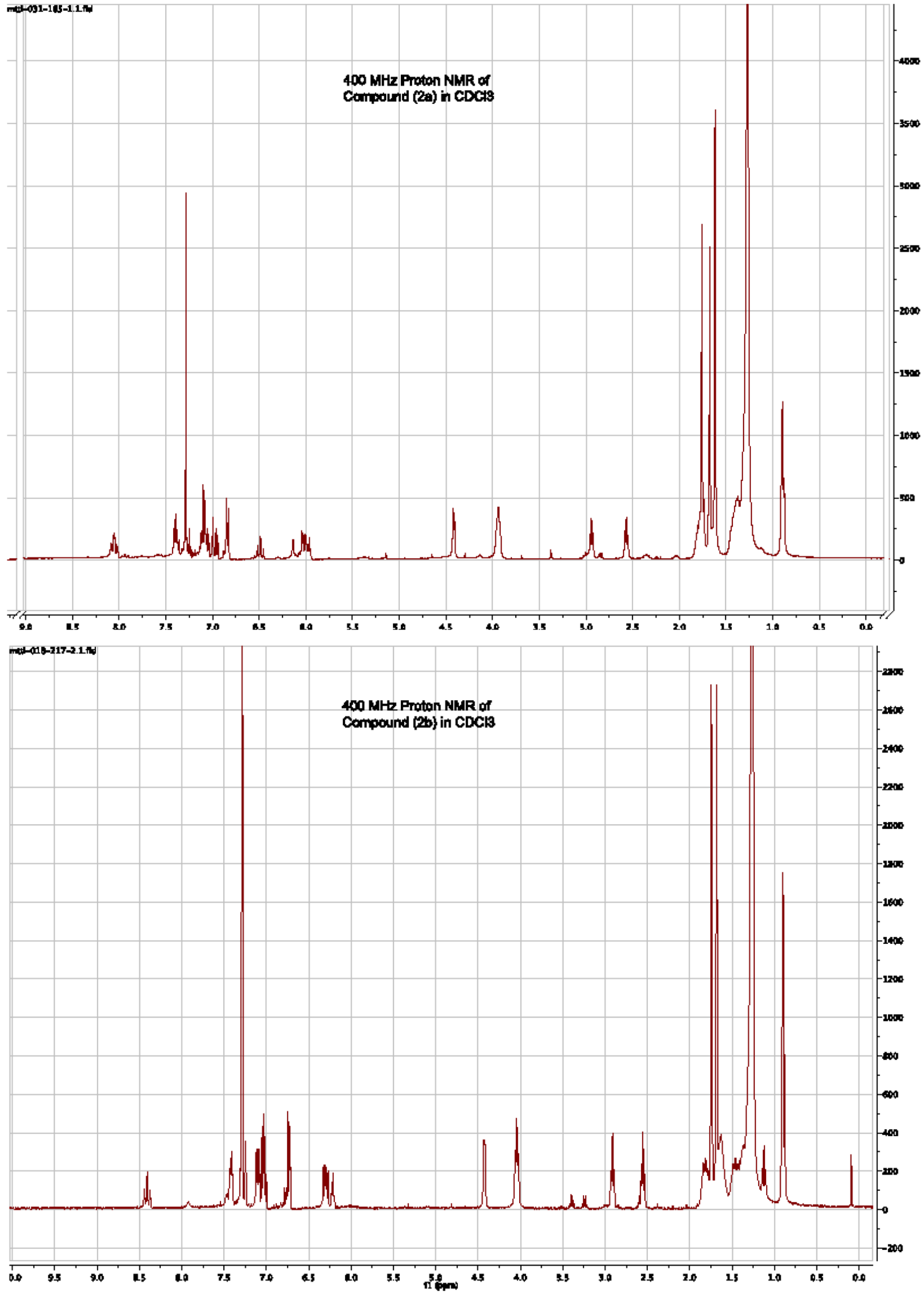

Supplementary Figure S1: NMR spectra of phenol-substituted lipophilic reporters. A. Compound 2a; 400MHz proton NMR ($CDCl_3$): 8.05 (m, 2H), 7.38 (m, 2H), 7.20(m, 4H), 7.05 (m, 2H), 6.94-7.00 (m, 2H) 6.84 (m, 2H), 6.50 (m, 1H), 6.14 (m, 1H), 6.0 (m, 1H), 5.96 (m, 1H), 4.40 (d, 2H), 3.94 (m, 4H), 2.96 (m, 2H), 2.57 (m, 2H), 1.85-1.60 (m), 1.5-1.2 (m), 0.89 (t, 6H). **B.** Compound 2b; 400MHz proton NMR ($CDCl_3$): 8.41 (t, 1H), 7.42 (m, 2H), 7.10(m, 2H), 7.03 (m, 2H), 6.75 (d, 2H), 6.30 (m, 2H), 6.20 (m, 1H), 6.00 (m, 1H), 4.40 (d, 2H), 4.05 (m, 4H), 2.91 (m, 2H), 2.55 (m, 2H), 1.90-1.60 (m), 1.5-1.2 (m), 0.90 (t, 6H).

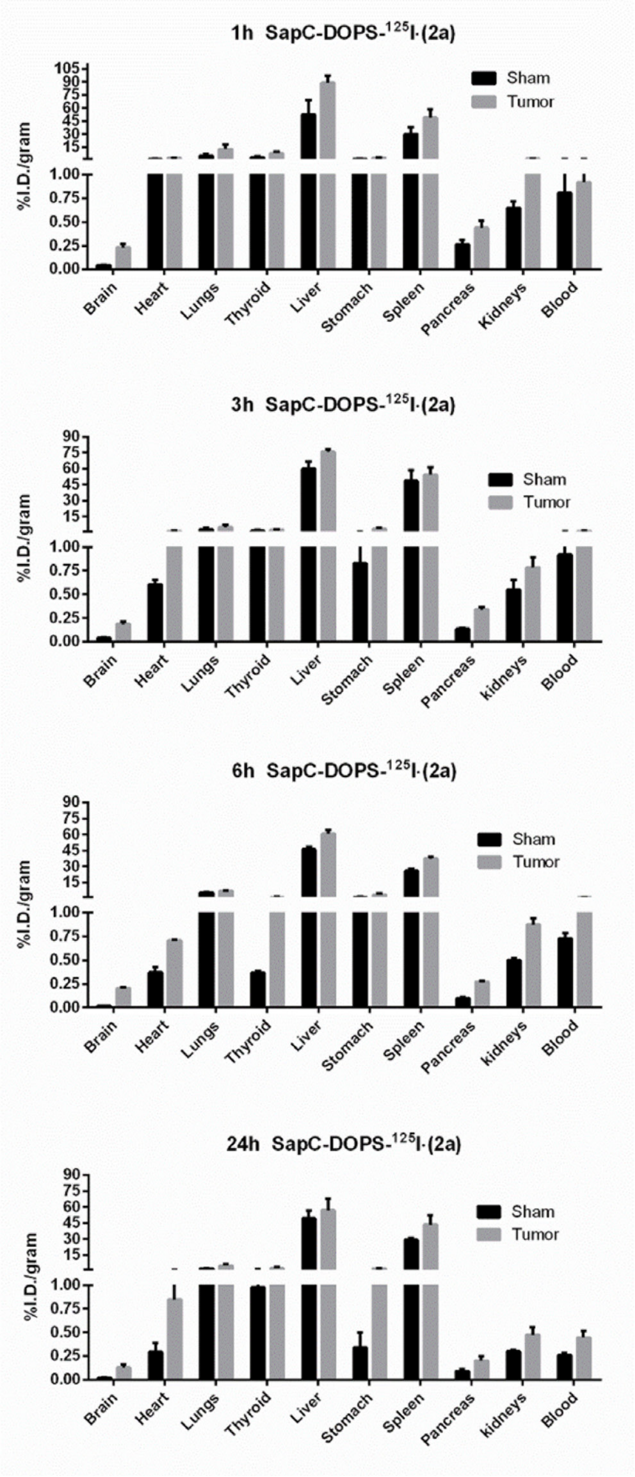

Supplementary Figure S2: Tissue biodistribution of SapC-DOPS-^{125}I· (2a). Tissue radioactivity was measured with a gamma counter at different time points after i.v. injection with radiolabeled SapC-DOPS (150 µl; 6.03 ± 0.02 µCi/).
